# Genetic, molecular and physiological basis of variation in *Drosophila* gut immunocompetence

**DOI:** 10.1038/ncomms8829

**Published:** 2015-07-27

**Authors:** Maroun S. Bou Sleiman, Dani Osman, Andreas Massouras, Ary A. Hoffmann, Bruno Lemaitre, Bart Deplancke

**Affiliations:** 1 Global Health Institute, School of Life Sciences, Station 19, EPFL, 1015 Lausanne, Switzerland; 2 Institute of Bioengineering, School of Life Sciences, Station 19, EPFL, 1015 Lausanne, Switzerland; 3 School of BioSciences, Bio21 Institute, The University of Melbourne, Parkville, Victoria 3010, Australia; 4 Swiss Institute of Bioinformatics, 1015 Lausanne, Switzerland

## Abstract

Gut immunocompetence involves immune, stress and regenerative processes. To investigate the determinants underlying inter-individual variation in gut immunocompetence, we perform enteric infection of 140 *Drosophila* lines with the entomopathogenic bacterium *Pseudomonas entomophila* and observe extensive variation in survival. Using genome-wide association analysis, we identify several novel immune modulators. Transcriptional profiling further shows that the intestinal molecular state differs between resistant and susceptible lines, already before infection, with one transcriptional module involving genes linked to reactive oxygen species (ROS) metabolism contributing to this difference. This genetic and molecular variation is physiologically manifested in lower ROS activity, lower susceptibility to ROS-inducing agent, faster pathogen clearance and higher stem cell activity in resistant versus susceptible lines. This study provides novel insights into the determinants underlying population-level variability in gut immunocompetence, revealing how relatively minor, but systematic genetic and transcriptional variation can mediate overt physiological differences that determine enteric infection susceptibility.

## Abstract (web summary)

Animals rely on a multitude of resistance and tolerance mechanisms to resist harmful gut microbes. Here, the authors explore the genetic, molecular and physiological basis underlying the remarkable phenotypic variation in resistance to enteric bacterial infection in *Drosophila melanogaster*.

## Graphical abstract

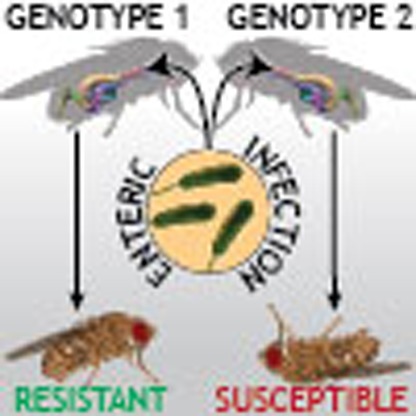

Given the constant exposure to potentially harmful pathogens, gut-bearing organisms developed an ensemble of molecular and cellular processes that together constitute ‘gut immunocompetence'^1^,^2^,^3^. Phylogenetically distant species share similarities in innate immune pathways^4^ and major structural and physiological gut features^5^,^6^. The study of gut immunocompetence in one system can therefore shed light on general aspects throughout the phylogeny. In *Drosophila melanogaster*, great strides have been made in elucidating the biological processes underlying gut immune defence. Notably, studies in the fly gut revealed that enteric infection leads to an intricate interplay between immunological, stress and repair mechanisms^7^,^8^,^9^,^10^,^11^. After oral ingestion, Gram-negative bacteria are able to induce the production of antimicrobial peptides and reactive oxygen species (ROS) by the enterocytes, which neutralizes the infectious microbes but also leads to tissue lesions^12^,^13^. Damaged intestinal cells then release cytokines leading to intestinal stem cell activation and tissue regeneration^9^,^14^,^15^.

How host genetic variation impacts these processes and how this is specifically encoded at the molecular and cellular levels is however still poorly understood, even though there are multiple examples where genetic variation affects an organism's susceptibility to infectious agents, including intestinal pathogens^3^. This may have far-reaching implications beyond acute disease. Indeed, the inability to effectively clear pathogens, to restrain the mounted immune response or to repair the damaged intestinal region may lead to chronic gut pathologies^16^. Elucidating the genetic and molecular determinants that mediate variation in gut immunocompetence is therefore of critical importance.

To address this, we used *Drosophila* not only because it is quickly gaining importance as a useful model to study the aetiology of inflammatory bowel diseases^14^,^17^, but also since it allows the analysis of molecular and organismal traits in a physiologically relevant and highly accessible system. The use of inbred fly lines allows assessment of the impact of infection on distinct, but constant genetic backgrounds to tease out the effect of the genotype from environmental effects^18^,^19^,^20^,^21^,^22^,^23^,^24^. This ability has been previously exploited to examine naturally occurring variation in pathogen susceptibility at a systemic level^22^,^23^,^24^, albeit to our knowledge not yet in the gut. Specifically, we used the *Drosophila* Genetic Reference Panel (DGRP)^18^,^25^ to explore variability in gut immunocompetence-related parameters and aimed to decipher the molecular and physiological determinants driving them. We found striking variation in survival to enteric bacterial infection and identified key underlying genetic variants, transcriptional modules and physiological signals.

## Results

### Genetic variation in susceptibility to enteric infection

**Figure 1:**
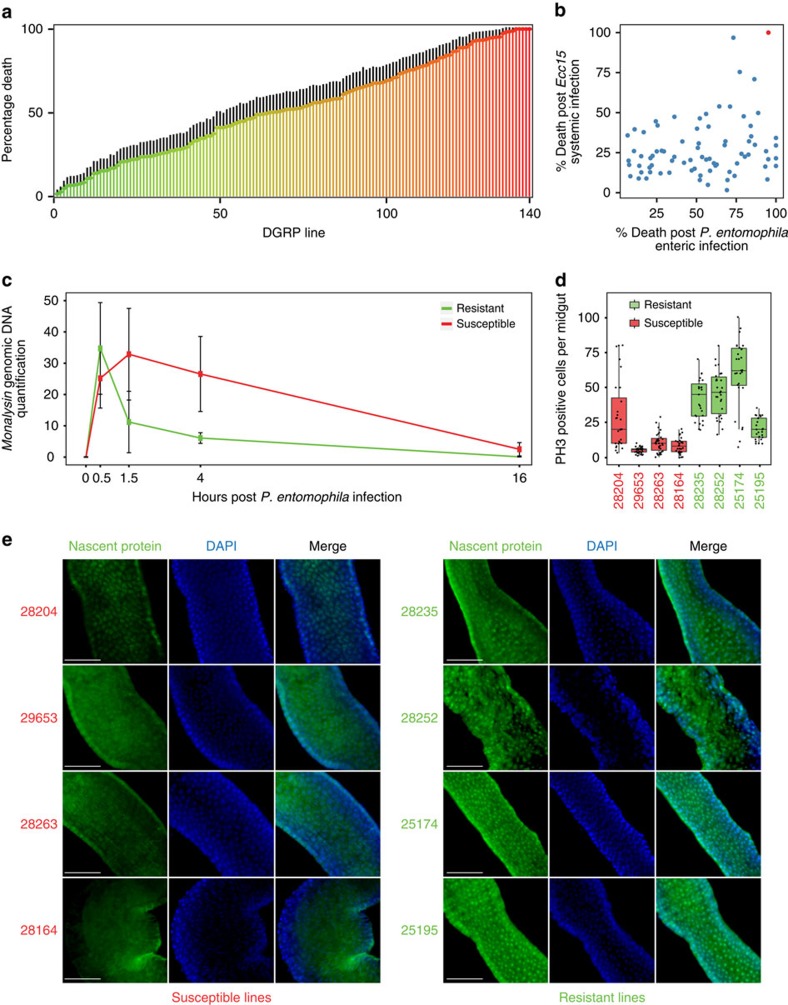
Susceptibility to infection is highly variable among DGRP lines and multifactorial. (**a**) Bar graph showing for each of the 140 DGRP lines (*x* axis) the percentage of dead female flies (*y* axis) 3 days post-enteric infection with *Pseudomonas entomophila* (*A* 100). Data shown are averages from three biological replicates (±s.e. of the proportion; *n*>60 females per line). (**b**) A scatter plot of 78 DGRP lines revealing an absence of correlation in proportion death between enteric (by 3 days post-*P. entomophila* ingestion) and systemic (by 10 days post-septic injury with *Ecc15*) infection. DGRP line 25745 (red) is highly susceptible in both conditions and features a rare mutation in the *dredd* gene. (**c**) Quantification of *P. entomophila*-specific *monalysin* genomic DNA by qPCR reveals differences in *P. entomophila* clearance between four susceptible and four resistant DGRP lines over time (ANOVA *P*=0.00343 for the effect of susceptibility class; see Supplementary Methods for details on statistics). (**d**) Quantification of PH3-positive cells per female midgut dissected 8 h post enteric infection with *P. entomophila* reveals that infected resistant lines have more mitotically active stem cells than those of susceptible lines (*n*>30 guts per line; ANOVA *P*<0.00001 for difference between susceptibility classes). (**e**) Measurement of the incorporation of a methionine analogue, L-azidohomoalanine (green staining), in the R2 region^56^ of the anterior midgut shows that susceptible lines are not able to synthetize proteins after infection in contrast to resistant lines. Note that while the same midgut region was sampled, no gross morphological differences in the shape or regionalization of the gut can be observed between resistant and susceptible flies after infection. However, this does not rule out subtle differences at the cellular level. Scale bar 50 μm.

To assess the extent of gut immunocompetence variation in genetically distinct individuals, we measured fly survival following enteric infection with the entomopathogenic bacterium *Pseudomonas entomophila*^26^ in 140 DGRP lines whose genomes have been comprehensively characterized for single nucleotide polymorphisms (SNPs) and non-SNP variants^18^,^19^,^27^. We found striking and reproducible variation in the DGRP lines' survival (Fig. 1a; Supplementary Fig. 1a; Supplementary Table 1), comparable to previous observations regarding natural variation in systemic immunity in *Drosophila*^23^. While around 50% of the tested lines harbour the natural endosymbiont *Wolbachia*^19^, this had no effect on susceptibility (Supplementary Fig. 1b). To eliminate the possibility that the differential susceptibility of the lines is due to differences in commensal bacteria^28^, we infected five lines randomly chosen from each phenotypic class (resistant or susceptible) in germ-free conditions. The loss of commensals did not alter their relative susceptibility, indicating that the endogenous microbiota do not majorly impact on susceptibility class (Supplementary Fig. 1c). We also evaluated whether our results could be biased by differences in feeding behaviour between DGRP lines but found no consistent difference in food uptake between resistant and susceptible lines (Supplementary Fig. 1d). To determine if this variability in survival is specific to enteric infection, we assessed susceptibility of DGRP lines to systemic infection with *Erwinia carotovora carotovora* 15 (*Ecc15*) (Fig. 1b). We did not use *P. entomophila* since it leads to very fast lethality in this condition, which renders the scoring of a meaningful phenotype difficult. We found little correlation between the two infection conditions and pathogens (Pearson correlation, *r*=0.23, *n*=78, *P*=0.0395). This observation suggests that the determinants of gut immunocompetence are distinct from those that govern systemic immunity^29^. However, one line, 25745, was highly susceptible in both infection conditions (Fig. 1b). We found that this fly line contains a null mutation in the *dredd* gene, a component of the immune deficiency (Imd) pathway required to resist Gram-negative bacterial infection^7^,^30^ (Supplementary Fig. 2a–d). Mutations with such a strong loss-of-function phenotype tend to be rare in a natural population and do not capture most of the underlying natural variation in gut immunocompetence^20^. For instance, the mutation we identified in *dredd* was found in only one of 205 genotyped DGRP lines^18^. Moreover, in a natural population, such a rare recessive allele would be mostly found in heterozygous form, which could explain why it has not been eliminated by purifying selection. We next examined whether the observed differences in survival is specific to *P. entomophila* by orally infecting DGRP lines with a clinical isolate of *Pseudomonas aeruginosa* (*PA14*). Specifically, using a similar infection protocol as for *P. entomophila* (Methods), we infected four randomly selected lines from the lower 10% in terms of survival to *P. entomophila* infection (that is, resistant) and four randomly from the upper 90% (that is, susceptible, excluding the *dredd* mutant line discussed above) and compared survival after 3 days (Supplementary Fig. 3). DGRP lines that were resistant to oral infection by *P. entomophila* were also resistant to *PA14*, while three of the four tested lines that were susceptible to *P. entomophila* were also susceptible to *PA14*. These results suggest that the DGRP phenotypes observed for *P. entomophila* infection may reflect a more general pattern in that they may be due to a common, likely bacterium-independent genetic and molecular mechanism that mediates oral infection susceptibility.

### Characterization of lines from the phenotypic extremes

We then assessed the dynamics of intestinal pathogen colonization and clearance in the same eight DGRP lines as used for the *PA14* infection experiment. Here, we quantified *P. entomophila* genomic DNA in fly guts at different time points post infection (Fig. 1c), providing new insights into the colonization behaviour of *P. entomophila* in the fly gut. Resistant and susceptible lines exhibited no significant difference in intestinal *P. entomophila* loads 30-min post infection, corroborating the results of the feeding assay. In addition, both classes of lines were able to clear *P. entomophila* from the gut after ∼16 h (Fig. 1c), suggesting that the impact of enteric infection with *P. entomophila* on survival is determined by the initial pathogen exposure and not persistence. Importantly, the rate of clearance was different between the two phenotypic classes with resistant lines reducing intestinal *P. entomophila* levels much faster than susceptible lines (analysis of variance (ANOVA) *P*=0.0033 for susceptibility class). This indicates that rapid eradication of *P. entomophila* as an immediate defence response could play a role in the final outcome of the infection. In *Drosophila* laboratory strains, *P. entomophila* infection causes severe irreversible intestinal epithelial damage in comparison to other pathogens^15^,^31^. Specifically, *P. entomophila*-induced inhibition of protein synthesis in the gut impairs both immune and repair programs leading to low epithelial renewal^31^. We examined whether the two DGRP phenotypic classes exhibit differences in protein synthesis and, as a consequence, variations in gut regenerative capability by measuring intestinal stem cell division, a quantitative readout of epithelial renewal. We found that guts of resistant lines are still able to translate proteins and induce a greater number of mitotic stem cells than those of susceptible lines (Fig. 1d,e). Collectively, our findings indicate that *P. entomophila* infection does not always lead to lethality caused by translation inhibition as previously suggested^31^, re-emphasizing the importance of host genetic background in determining the response to as well as outcome of infection.

### Genetic architecture of susceptibility to enteric infection

**Figure 2:**
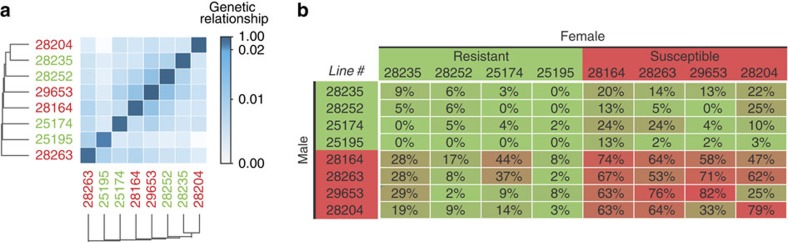
Gut immunocompetence is a largely additive, complex trait. (**a**) The genomic relationship matrix shows an absence of genetic relatedness among either resistant or susceptible lines respectively. (**b**) Percentage death for F1 flies in a full diallel cross between four susceptible and four resistant DGRP lines (by 3 days post-enteric infection with *Pseudomonas entomophila* (*A* 100)).

It is conceivable that physiological and survival differences between resistant and susceptible lines are a mere consequence of high genetic relatedness among lines from each phenotypic class. To explore this possibility, we used the available genetic relationship matrix for the eight DGRP lines (http://dgrp2.gnets.ncsu.edu/), but did not observe genetic clustering of phenotypic classes, as expected^18^ (Fig. 2a). However, a significant part of the observed variation in survival is due to genetic factors as the heritable component estimate is 0.61 (Methods). To gain insights into the genetic architecture of survival, we performed a complete diallel cross, where we generated all possible hybrid combinations by crossing the eight lines to each other. We then measured their susceptibility to *P. entomophila* infection. The F1 progeny from crosses between different resistant lines were resistant (Fig. 2b) and the F1 progeny from crosses between different susceptible lines were mainly susceptible, thus there was no evidence of consistent heterosis. The lack of resistance appearing in crosses between susceptible lines implies that susceptibility is not a mere consequence of inbreeding depression. Moreover, F1 progeny from crosses between resistant and susceptible lines tended to exhibit an intermediate susceptibility phenotype as expected when there are additive effects. Indeed, an analysis of the diallel cross data (Supplementary Table 2) revealed both additive effects reflected in general combining ability (ANOVA *P*=0.00001) and dominance effects reflected in specific combining ability (ANOVA *P*<0.00001)^32^. There were also various interactions between strains due to male and female parental combinations (Supplementary Table 2), suggesting that the extent of susceptibility depends on the specific combination of strains tested. In general, these patterns indicate that natural variation in survival to infection is partly additive, but also depends on the combination of strains being crossed, suggesting a complex genetic architecture.

### Genome-wide association study for survival to infection

**Figure 3:**
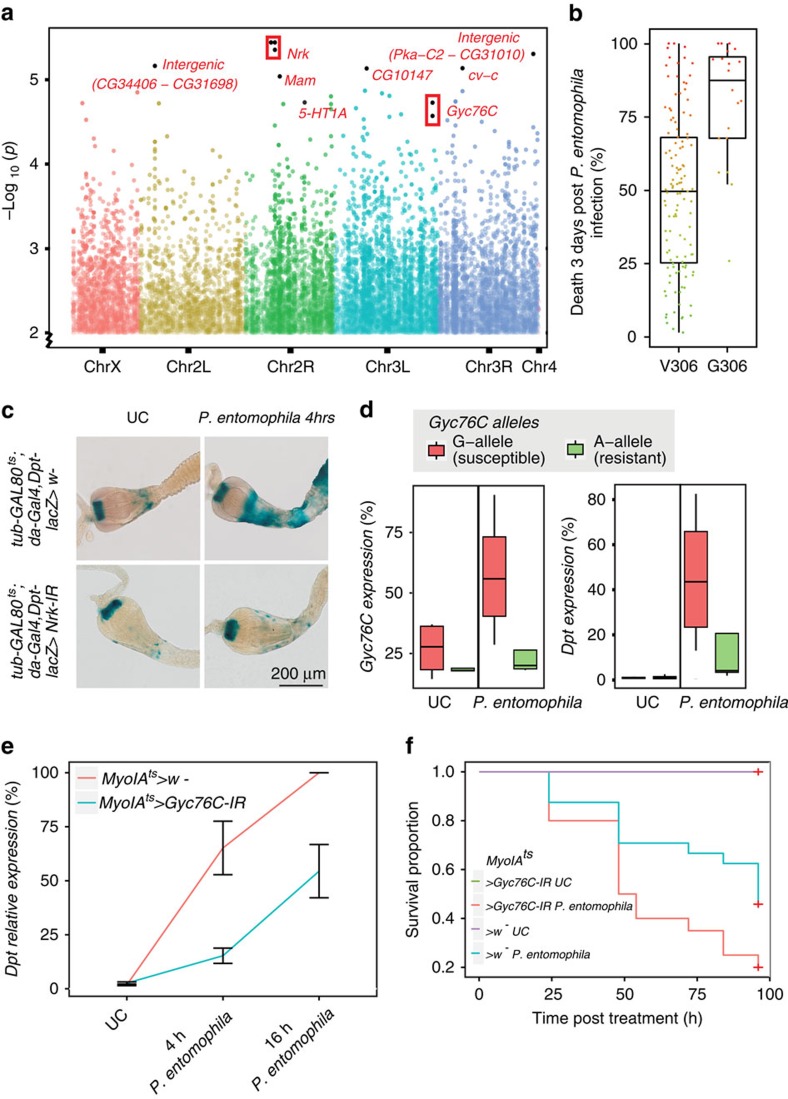
GWAS reveals genetic loci underlying susceptibility to infection. (**a**) Manhattan plot of the *P*-values (*y* axis) for the association between genomic variants in DGRP lines and *Pseudomonas entomophila* susceptibility. A non-parametric Kruskal–Wallis test was performed using proportion death at day 3 as phenotype. The *x* axis represents the genomic location. Multiple variants in a single gene are bounded by a box. (**b**) Susceptibility of DGRP lines grouped by the *Nrk* allele (GWAS *P*=3.6e−6) that changes the coding sequence at position 306 of the protein (at chr2R:9048897). Note that *Drosophila simulans*, *Drosophila sechelia*, *Drosophila yakuba*, and *Drosophila erecta* all have the variant G-allele. (**c**) Knockdown of the top GWAS hit, *Nrk*, using a ubiquitous driver (*da-gal4*) highly reduces the activity of the immune activation reporter *Dpt-lacZ* in the gut as revealed with X-Gal staining (*P. entomophila A* 50 was used to avoid the anticipated inhibition of translation effect of *P. entomophila* at *A* 100 (ref. 31)). UC=unchallenged flies. (**d**) RT–qPCR experiments on gut total RNA from females show that four susceptible DGRP lines harbouring the G-allele at the *Gyc76C* locus (chr3L:19769316) express *Gyc76C* at higher levels after *P. entomophila* infection, in comparison to resistant lines carrying the A-allele. *Dpt* transcript induction is higher in susceptible DGRP lines carrying the G-allele in *Gyc76C* (ANOVA *P* for allele effect in the challenged condition for *Gyc76C* and *Dpt* is 0.00205 and 0.0344, respectively). (**e**) *Gyc76C* knockdown in enterocytes using the thermosensitive *MyoIA-gal4* driver shows that *Gyc76C* regulates the induction of *Dpt* transcript in the gut 4h and 16 h post-*P. entomophila* infection (ANOVA *P*=0.00741 for line effect; error bars represent standard deviation around the mean of three replicates). (**f**) Survival analysis of females that are orally infected with *P. entomophila* shows a lower survival rate of *MyoIA*^*ts*^*>Gyc76C-IR* flies compared to wild type (Log-Rank test *P*=0.0351 for comparison between *Gyc76C* knockdown and wild type in challenged condition). **d**–**f** data is based on at least three independent biological replicates.

To uncover genetic determinants underlying variation in immunocompetence, we performed a genome-wide association study (GWAS) on survival using both a non-parametric (Fig. 3a) and parametric test (Supplementary Fig. 4a). Unlike a previous study dealing with survival to viral infection in DGRP lines in which one quantitative trait locus (QTL) with large effect was identified^24^, we obtained 27 QTLs at an arbitrary *P*-value of 10^−5^, even though there was no clear point of departure from expectations in the *Q*–*Q* plot (Supplementary Fig. 4b). The results were largely consistent between both GWAS analysis procedures and a maximum of 19% of the phenotypic variance could be explained by a single QTL (Supplementary Table 3). The small sample size and the truncated distribution from which QTLs are chosen to estimate effect sizes can result in an overestimation of the proportion of variance explained, a phenomenon known as the ‘Beavis effect'^33^. This could be further exacerbated by linkage between SNPs (Supplementary Fig. 4a). To account for redundancy between linked SNPs, we also performed an iterative multiple-SNP regression^34^. Interestingly, as few as four SNPs can explain ∼50% of the phenotypic variance (Supplementary Table 4). Moreover, we performed a permutation analysis to evaluate the Beavis effect. In short, we sampled groups of lines of different sizes, ranging from 70 to 140, and performed multi-SNP regression. For each sample size, we performed 100 permutations with random resampling (Supplementary Fig. 5). We found that the proportion of variance explained, *R*^2^, decreases as the sample size increases, as expected, yet starts levelling-off at larger sample sizes, suggesting that the correct proportion of variance accounted by the SNPs is being approached at the larger sample sizes.

The most significant QTLs were located in the neurospecific receptor kinase (*Nrk*) gene, which belongs to an evolutionarily conserved stress-response network from *Drosophila* to mammals^35^. One of the three linked *Nrk* QTLs (Supplementary Table 3), which explains 14% of the phenotypic variance, is a non-synonymous polymorphism (*P*=3.6 × 10^−06^) in residue 306 of the protein (G or V). The minor allele (15% frequency) appears to be the ancestral allele since it is found in the four closest sequenced *Drosophila* species. Interestingly, lines harbouring this minor allele were mainly susceptible (Fig. 3b). To test if *Nrk* affects the antibacterial immune response, we measured the activity of the Imd pathway reporter *Diptericin-lacZ* (*Dpt-lacZ*)^12^ in wild-type and *Nrk* knockdown flies. In contrast to infected control guts, where *Dpt-LacZ* reporter was induced in the cardia and anterior midgut, *Nrk* knockdown flies have markedly reduced *Dpt-lacZ* activity (Fig. 3c). We also investigated the knockdown effect of several other genes that harboured strong QTLs with *Gyc76C* producing the most robust and greatest reduction in *Dpt-lacZ* activity (Supplementary Fig. 6c). *Gyc76C* contains a QTL (*P*=1.86 × 10^−05^) that explains 15% of the variance (Supplementary Table 3), and has recently been described as a modulator of the Imd pathway in response to salt stress in the Malpighian tubules^36^. Susceptible DGRP lines carrying the G-allele of the QTL expressed *Gyc76C* at higher levels than resistant lines (A-allele) post infection (Fig. 3d). Remarkably, endogenous *Dpt* transcript induction followed a similar trend (Fig. 3e). Knocking down *Gyc76C* expression specifically in enterocytes of adults also showed that *Gyc76C* diminishes *Dpt* induction (Fig. 3e) and reduces fly survival after enteric infection (Fig. 3f). Since Gyc76C is a membrane receptor capable of the activation and nuclear translocation of the Imd transcription factor Relish^36^, it may activate the Imd pathway in the gut independent of PGRP-LC, the canonical Imd pathway receptor. Taken together, these results suggest that our GWAS identified at least two novel genes that are capable of modulating gut immunocompetence and that were not previously implicated in canonical gut immune response pathways.

### Transcriptomic analysis of phenotypic extremes

**Figure 4:**
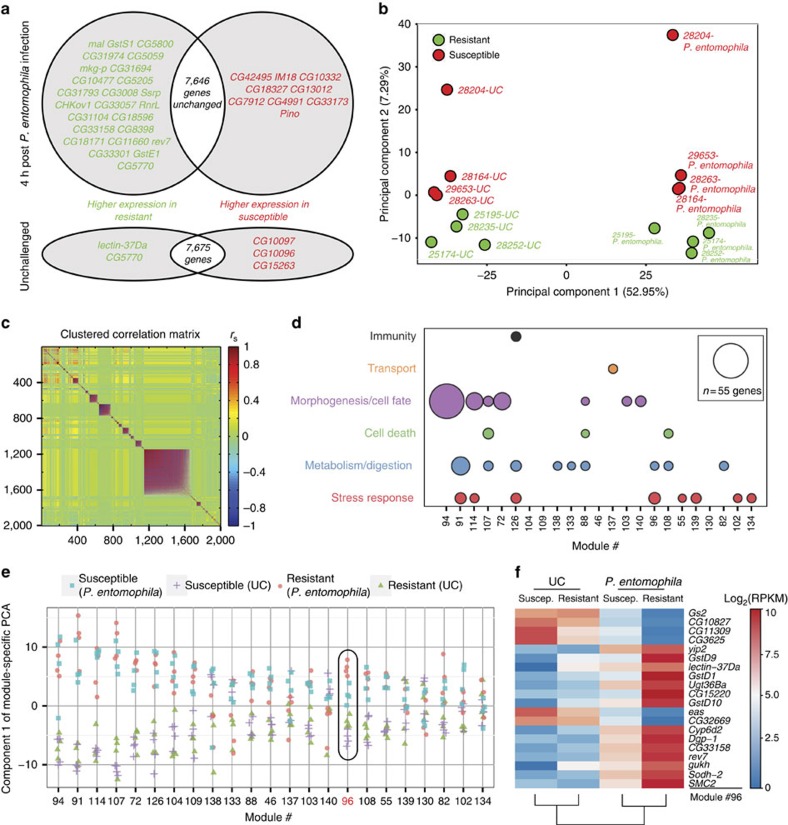
Specific gene expression signatures define susceptibility to bacterial enteric infection. (**a**) Venn diagram showing differentially expressed genes (as revealed by RNA-seq experiments) between four resistant and four susceptible DGRP lines, in the unchallenged condition and 4 h post *Pseudomonas entomophila* infection (*q*-value<0.2, two-fold change). Genes in red and green have higher levels in susceptible and resistant lines respectively. The number of genes (black) indicated in the intersections represents the total number of non-differentially expressed genes. (**b**) Principal component analysis (PCA) on the top 2,000 varying genes between the 16 samples reveals that resistant lines cluster separately from susceptible lines, before (*UC*) and post-*P. entomophila* infection. PC1 separates samples based on treatment whereas PC2 separates them based on susceptibility class. (**c**) Modulated modularity clustering analysis on the top 2,000 varying genes identifies 24 correlated transcriptional modules (*n*≥15 genes). Each coloured point represents the spearman correlation (*r*_s_) between two genes. (**d**) A selection of functional categories identified by gene ontology (GO) analysis of genes belonging to the different modules identified in **c** (excluding the largest module with *n*=523, Supplementary Data 3). For the GO analysis, we used the Database for Annotation, Visualization, and Integrated Discovery (DAVID). (**e**) PCA using the expression levels of genes within each of the 24 modules identifies module #96 as the only module for which the lines are clearly separated on the first principal component according to treatment and susceptibility. (**f**) Heat map of gene expression levels in module #96 reveals important differences across susceptibility classes and treatment conditions.

Variability in survival and physiology among DGRP lines could in part be explained by system-specific transcriptional differences. We therefore performed RNA-seq on 16 gut samples comprising the same four susceptible and four resistant lines as introduced above in the unchallenged condition and 4 h after *P. entomophila* infection (Supplementary Fig. 7a). Genes (1287) were differentially expressed 4 h post infection compared with the unchallenged condition when all eight lines were treated as replicates (false discovery rate (FDR) adjusted *P*-value<0.05 and two-fold change, Supplementary Data 1). This set of genes overlaps with what we have previously shown when characterizing the gut transcriptional response to *P. entomophila* infection, even though that analysis was carried out using microarrays and on a different genetic background (*Oregon*^*R*^)^31^. However, when we looked for differences in gene expression between the four resistant and four susceptible lines by pooling the samples of each susceptibility class, very few genes exhibited significant differential gene expression. Specifically, the expression of only 5 and 34 genes were changed in the unchallenged and challenged guts, respectively, when comparing phenotypic classes (Fig. 4a; Supplementary Data 2). This may reflect reduced statistical power given the large number of genes that are compared. In addition, it is possible that small but systematic differences in gene expression collectively differentiate resistant from susceptible profiles. We therefore performed principal component analysis (PCA) on 2000 genes with the highest expression variance in the 16 transcriptomes. Since infection status has a large impact on the transcriptome, expression profiles derived from infected samples were separated from those of unchallenged samples on the first principal component (PC), which explains 53% of the variance (Fig. 4b). Strikingly, even before infection, profiles of resistant lines were separated from those of susceptible lines based on the second PC, which explains 7.3% of the variance (Fig. 4b). This separation implies that the basal intestinal transcriptional state of resistant lines is distinct from that of susceptible lines, which may either define or reflect a molecular pre-disposition to enteric infection susceptibility. To dissect the molecular signatures that underlie this transcriptional stratification of the two phenotypic classes, we performed modulated modularity clustering^37^ on the same 2000 genes. We identified 24 transcriptional modules including >15 correlated genes (Fig. 4c; Supplementary Data 3). On the basis of Gene Ontology analysis and manual annotation^38^, we assigned the genes within the modules to six functional groups (Fig. 4d). To identify those modules whose gene levels clearly separate the lines according to treatment and phenotypic class, we systematically performed PCA on each module by taking the expression levels of its genes (Fig. 4e). We found that in module #96, samples are clearly separated on the first PC, even though the probability for such a separation to spuriously occur is <3 in 10,000 (Fig. 4e; Supplementary Fig. 7b,c). This module contains 20 genes, of which nine are related to stress response and most notably to ROS metabolism (Fig. 4e,f) and collectively explains 29% of the observed phenotypic variation (Supplementary Table 5). Other modules such as #102 (16 genes) also separated the samples on the first two PCs (Supplementary Fig. 8). Interestingly, module #102 likewise contains several ROS-related genes such as *Cyp6a9* and *Thioredoxin-2* (*Trx-2*)^39^. ROS are essential signalling molecules and immune effectors that are induced by the infected gut to neutralize pathogens^13^ and promote intestinal renewal^14^. However, a high ROS load can also cause inhibition of protein translation and consequently severe intestinal damage^31^, necessitating a finely tuned regulation of ROS production and metabolism^40^.

### A role for ROS in variation in susceptibility

**Figure 5:**
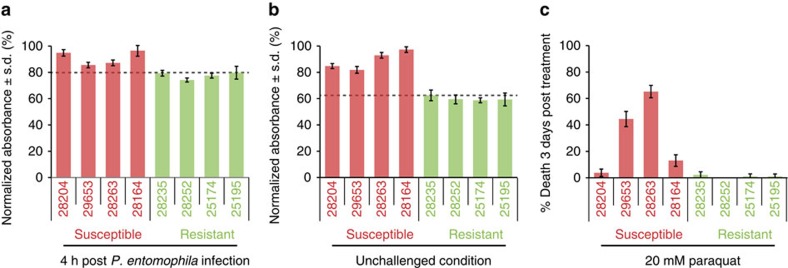
Diversity in ROS metabolism is a feature of variable susceptibility. (**a**–**b**) Measurement of ROS activity in flies before and after *Pseudomonas entomophila* infection reveals lower ROS levels in resistant compared with susceptible DGRP lines (mean normalized absorbance ± s.d., *n*=5 females per line and replicate, three replicates, ANOVA *P*<0.0001 for difference between susceptibility classes in both conditions). The dashed line marks the maximum level in resistant lines. (**c**) Percentage of dead female flies 3 days after Paraquat treatment. Percentages are averages from three experiments (± s.d., *n*>60 females/line, ANOVA *P*<0.0001 for difference between susceptibility classes).

To investigate the physiological relevance of ROS in mediating inter-individual differences in gut immunocompetence, we compared ROS levels in resistant versus susceptible lines (Fig. 5a,b). Importantly, ROS levels were significantly lower in resistant lines in both conditions (ANOVA *P*=2.98 × 10^−7^ for susceptibility class in unchallenged condition and *P*=1.43 × 10^−11^ in challenged condition). This may reflect a more efficient ROS metabolism in resistant lines, possibly mediated by the higher expression levels of the majority of genes in the focal module #96 compared with susceptible lines (Fig. 4f). Since too much ROS inhibits translation and epithelial renewal resulting in lethality^31^, it appears that resistant lines utilize ROS in a more effective and less noxious manner than susceptible lines (Fig. 1c,e). To investigate this hypothesis, we evaluated the survival of the same lines to ingestion of paraquat, a ROS-catalyzing chemical reagent. Most susceptible lines showed higher lethality compared with resistant lines (Fig. 5c), supporting the role of ROS as one of the principal components underlying variation in gut immunocompetence.

## Discussion

Direct exposure to environmental insults such as pathogens has driven the alimentary canal to establish numerous protective and homeostatic mechanisms^28^. Considerable efforts have been invested in characterizing mechanisms underlying intestinal immunity using model organisms like *Drosophila*. However, most of these studies identified genes with large effects involved in canonical immune pathways^7^. The aim of our study was to go beyond these classical analyses to uncover first of all the extent of inter-individual variation in gut immunocompetence and in a subsequent step the underlying genetic and molecular determinants. We found striking differences in the overall susceptibility to enteric infection, not only in survival, but also in related physiological aspects including bacterial load, stem cell activity and infection-induced inhibition of translation. A first important implication of these findings is that the outcome of classical *Drosophila* genetics experiments involving standard laboratory strains may not always be generalizable to all wild-type strains. Indeed, while the use of such standard strains is valuable to increase reproducibility, a downside is that it may lead to conclusions that are only true in specific genetic backgrounds^41^,^42^ as we demonstrate here for pathogen-induced inhibition of translation (or lack thereof) in DGRP lines.

This phenomenon likely reflects the inherently complex nature of traits like gut immunocompetence since they are the result of the interplay of many biological processes, each of which could be affected by many genomic loci with small to medium effects. The results from our GWAS analysis are consistent with this hypothesis as they suggest that relatively common alleles located in various parts of the genome drive gut immunocompetence in additive manner. If rare variants resulted in reduced survival to infection in susceptible lines, then crossing two susceptible lines should have resulted in a resistant hybrid. Moreover, deleterious mutations affecting gut immunocompetence could be under strong purifying selection, further reinforcing a genetic architecture of multiple loci with relatively small effects^43^,^44^.

A consequence of such a genetic architecture is that it renders the prediction of a trait from genotypic information difficult. An attractive approach to improve phenotypic predictions is the complementation of genetic data with *in vivo* measurements of molecular parameters since the latter may yield mechanistic insights that may not be immediately obvious from GWAS analyses, which, similar to our study, are often performed on rather coarse-grained phenotypic read-outs (such as survival here)^45^. Our finding that the transcriptomes of resistant and susceptible extremes can be separated by PCA even before infection is interesting in this regard, as it suggests that there are systematic molecular differences underlying susceptibility to enteric infection. This observation also implies that with a large enough sample size, signatures of susceptibility could be mined from the data for both a better biological understanding and prediction of gut immunocompetence. In this study, we provide a proof of concept by clustering correlated transcripts into modules and identifying a candidate module linked to ROS metabolism. While the involvement of ROS in intestinal infection and homeostasis has been previously established^9^,^13^,^28^,^31^,^46^, it is particularly intriguing that it may also be one of the important factors that either mediate (or reflect) naturally occurring variation in gut immunocompetence, since lines from the phenotypic extremes contained significantly different intestinal ROS levels even before infection and reacted distinctly after exposure to the ROS-inducing chemical paraquat. As such, ROS levels, which are an indirect measure of stress, may have phenotype-predictive value, irrespective of whether differential ROS levels are a cause or a consequence of differences in gut immunity. Better utilization of ROS by the resistant lines may then constitute a tolerance rather than an active resistance mechanism^47^. But clearly, alleles for low tolerance have persisted in the population and we speculate that the underlying mechanisms could be conceptually similar to variation in immunity, where environmental heterogeneity and fitness trade-offs limit the effect of natural selection^48^.

Since enteric infection has a major impact on human and animal health, resolving the genetic and physiological contributions underlying continuous variation is of great importance. This is particularly the case in the developing world where almost 20% of child deaths can be linked to a pathogenic invasion of the intestine^49^. In many cases, this invasion is by opportunistic pathogens on immunocompromised individuals, who might have a functioning innate immune system like AIDS patients^50^. In addition, enteric infections by opportunistic *Pseudomonas* species have been reported in hospitalized patients^51^,^52^. Understanding the role of genetic variation in innate immunity could therefore shed more general light on susceptibility to opportunistic pathogens^53^ including members of the *Pseudomonas* genus^52^. Our study now reveals that identifying causal factors may present a substantial challenge in that the observed, overt physiological differences between resistant and susceptible lines appear to be driven by multiple genetic effects. We therefore postulate that a promising strategy could be the identification of transcriptional modules as informative biomarkers of disease susceptibility given their inherent dependence on the interaction between a genome and its environment. Alternatively, since transcriptome analyses are expensive diagnostic tools, knowledge gained from the study of transcriptional modules could be used in the discovery of novel biomarkers. Such insights into the molecular determinants of gut immunocompetence may help in developing control programs in invertebrate disease vectors as well as in better understanding the mechanisms underlying variability in susceptibility to enteric infections in human populations.

## Methods

### Fly stocks

DGRP lines were obtained from the Bloomington Stock Center and reared at room temperature on a standard fly medium. The fly medium recipe that we used is the following (for 1 l water): 6.2 g agar powder (ACROS N. 400400050), 58.8 g Farigel wheat (Westhove N. FMZH1), 58.8 g yeast (Springaline BA10), 100 ml grape juice, 4.9 ml propionic acid (Sigma N. P1386), 26.5 ml of methyl 4-hydroxybenzoate (VWR N. ALFAA14289.0) solution (400 g/l) in 95% ethanol and 1 l water. For RNAi (IR) studies, F1 progeny carrying one copy of the *da-Gal4* or *MyoIA-Gal4* with *tub-Gal80*^*ts*^ transgenes (and *Diptericin-lacZ* reporter in the case of *da-Gal4*) as well as one copy of *UAS-IR* (all in the *w*^*1118*^ background) were kept at 18 °C for 3 days post eclosion, and then moved to 29 °C for 8 days to activate the *UAS-IR*. The *UAS-Gyc76C-IR* line is a gift from Julien Dow, the *UAS-Nrk-IR* (CG4007 R2 and R3) fly lines were obtained from the DGRC stock centre. Imd pathway mutants used are *Dredd*^*B118*^ (ref. 30) and *Relish*^*E20*^ (ref. 54).

### Infection, paraquat treatment and survival experiments

*P. entomophila* L48 is a strain isolated from a female *D. melanogaster* fly collected at the Island of Guadeloupe^26^. *Ecc15* was obtained from the French Collection of Phytopathogenic Bacteria (INRA, Angers, France). *P. entomophila* and *Ecc15* were cultured in Lysogeny broth (LB) medium at 29 °C overnight. *P. aeruginosa* clinical isolate *PA14* (UPR 9022, Strasbourg) was cultured in Brain Heart Infusion broth at 37 °C overnight. For enteric infection, 3–5 day old females were first starved 2–3 h at 29 °C, and then transferred into vials with fly medium covered with filter disks soaked in a mix of bacterial pellet at OD_600 nm_ of 100 and 1.5% sucrose. For survival analysis, flies were transferred onto a fresh fly medium 24 h post infection, and maintained on a fresh and healthy medium during the survival assay. For Paraquat treatment, the same procedure as oral infection was followed except for the addition of a solution of 20 mM Paraquat dichloride hydrate (FLUKA Analytical #36541) in 1.5% sucrose instead of the bacterial pellet. For systemic *Ecc15* infection, adult flies were pricked in the thorax with a tungsten needle that had been dipped into a concentrated bacterial pellet with an OD_600 nm_ of 200.

### RT–qPCR

Total RNA was extracted from 20 guts including the crop, the cardia and the midgut using TRIzol reagent (Invitrogen). Malpighian tubules were removed from the samples. cDNA was then synthesized from 1 ug total RNA using *SuperScript II* enzyme (Invitrogen). quantitative PCR experiments were performed with a LightCycler 480 machine and the SYBR Green I kit (Roche). Relative gene expression was calculated after normalization to the control *RpL32* mRNA. Given the polymorphic nature of the DGRP lines, we assured that the primers did not target sites with polymorphisms. The primer sequences are available in Supplementary Table 6.

### Bacterial load measurement

Flies were orally infected with *P. entomophila* and then transferred to a fresh medium 30 min post infection. The DNA fractions were then isolated at indicated time points using the TRIzol manufacturer's protocol (Invitrogen). The bacterial load quantification was then assessed by quantitative PCR with *P. entomophila monalysin*-specific primers^55^ (Supplementary Table 6). Normalization has been performed on the host *RpL32* DNA.

### Assessment of nascent protein synthesis

To assess the levels of protein translation in susceptible and resistant guts, we used the Click-iT AHA (L-azidohomoalanine) for Nascent Protein Synthesis commercial kit (Invitrogen). Flies were orally infected for 16 h as described above, but by adding AHA reagent at 50 μM as final concentration to the infection mix. Guts were then dissected in 1X PBS Triton 0.3%, fixed for a minimum of 30 min in PBS 4% paraformaldehyde, and finally washed with PBS Triton 0.3%. DAPI reagent (Sigma) was used to stain DNA. The R2 region^56^ of the gut was visualized with an Axioplot imager (Zeiss).

### PH3 staining

Guts were dissected in Grace's insect medium (life technologies) and fixed for 15–20 min in PBS 4% paraformaldehyde. They were subsequently washed in PBS 0.1 triton (PBT), blocked in PBT 0.1% BSA (PBTA) for 1 h, and then incubated 2 h at 4 °C with primary and secondary antibodies in PBTA. Antibody used was 1/500 rabbit anti-PH3 (Millipore), 1/500 Alexa-594 anti-rabbit (Life Technologies).

### ROS measurement

To assess homeostatic ROS level as well as *P. entomophila*-induced ROS, we used the Amplex Red reagent (Invitrogen #A12222) as described previously^46^, by incubating six flies of each genotype with 100 μl of reaction buffer (pH 7,4) and 0.25 unit ml^−1^ of horseradish peroxidase (Sigma) for 1 h at 37 °C. The fluorescence was measured in a microplate reader at 550 nm.

### Genome-wide association analysis

We performed two GWAS. The first was performed on angle transformed proportion death at day 3 using PLINK v1.07 (ref. 57). Specifically, means of three repeats per line were taken as phenotype, and only biallelic SNP markers were considered. We calculated empirical *P*-values by using default adaptive permutation settings. The other GWAS was performed directly on the proportion data using a non-parametric Kruskal–Wallis one-way ANOVA by ranks test. In this pipeline, all variants can be considered, including non-SNPs, even if they are not biallelic. Specifically, we grouped overlapping variants for each line, creating a list of loci with two or more alleles in the population with a minimum allele count of 10. We then grouped the phenotypic measurement according to the allele of its line and performed a Kruskal–Wallis test. For each variant, 1,000 permutations of the phenotype data were performed to estimate the false discovery rate. Since our GWAS hits are of marginal significance, the false discovery rate within this range of *P*-values is high (for example, at *P*-value≤2e−05, the FDR is 0.66). Nevertheless, the two approaches yielded very similar candidate lists. For the multiple-SNP GWAS, please refer to the legend in Supplementary table 4.

### RNA-seq analysis

Four resistant (Bloomington 28235, 28252, 25174 and 25195) and four susceptible DGRP lines (Bloomington 28164, 28263, 29653 and 28204) were selected for RNA-seq experiments. These eight lines were infected 4 h with *P. entomophila* as indicated above, in parallel, the same eight lines were kept on 1.5% sucrose as controls. Twenty-five guts for each of the 16 samples were dissected and subsequent TRIzol RNA extraction was performed. We chose the 4 h post infection time point for multiple reasons. First, we have previously shown that major changes occur in the transcriptome as early as 4 h post infection. Importantly, these changes are not restricted to immediate immune responses, but extend to the homeostatic mechanisms like intestinal stem cell-induced regeneration and repair. So we reasoned that differences between resistant and susceptible lines could be resolved by that time. Another motivation for this choice stems from the fact that *P. entomophila* does not persist in the gut, and therefore, resistant lines could return to an uninfected state relatively quickly. In addition, fly mortality is still low to non-existent at 4 h post infection in susceptible lines. Libraries were prepared using the Illumina Truseq RNA kit and sequenced for 100 cycles on the Illumina HiSeq 2000 in the University of Lausanne Genomic Technologies Facility. Post processing was performed using Casava 1.82. There was an average of 25 million reads per sample. Reads were mapped to individual DGRP-predicted transcriptomes^19^. Count data was normalized using the Voom package in R. Each gene's reads per kilobase per million mapped reads (RPKM) value was calculated by averaging the RPKM values of its associated transcripts. Analysis of differential expression was performed using limma^58^. Gene RPKM values were used to perform principal component analysis using the FactoMineR package. Modulated modularity clustering was performed as in ref. 20 on the RPKM values of the 2,000 genes with the largest variance. We used the R built-in heatmap function with default settings for mean gene expression levels by phenotypic class in module #96.

### Quantitative genetic and statistical analyses

All statistical analyses were performed in R version 3.0.2 unless otherwise noted. We used angular transformation on percentage death data in all parametric analyses. For calculating the heritable component, we treated the transformed percentage death at day 3 as a Gaussian response in a random effects model of the form Y=*μ*+*L*+*R*+*ɛ* where *μ* is the mean proportion death of all lines, *L* is a random variable representing deviation of each line from the mean, *R* is a random variable representing the deviation of each line's biological replicate from the line mean, and *ɛ* is the residual error. We assumed that all variation is additive and that there is no epistasis and estimated the heritable component as *V*_A_/*V*_A_+*V*_E_, where *V*_A_ is the additive genetic variance and is equal to half the between-line variance, *V*_L_, since the lines are almost entirely homozygous and *V*_E_ is the environmental variance such that *V*_E_=*V*_R_+*V*_*ɛ*_. To estimate the proportion of variance accounted for by a certain QTL, we calculated *R*^2^ by performing linear regression taking the SNPs as factors. Pearson's product moment correlation between oral infection and septic injury was performed on the angular transformed line means between oral infection at day 3 and septic injury at day 10. For the bacterial load experiment, we analysed log_2_ relative ratios to *Rpl32* values using ANOVA where the line was nested in susceptibility class and treated as a fixed effect, time post infection was treated as a fixed effect, and experimental replicate was treated as a random effect. Nested ANOVA, where line is nested within susceptibility class, was used to compare the log_2_ transformed PH3 counts of the susceptibility classes. For the analysis of the effect of RNAi knockdown of *Gyc76C* on *Dpt* induction, ANOVA was used with genotype and time post infection as fixed effects and experimental replicate as a random effect. Separate nested ANOVA by condition was used to determine the effect of susceptibility class on ROS levels (normalized absorbance) where line was nested in susceptibility class and treated as a fixed effect and experimental replicate was treated as a random effect. We used the R built-in heatmap function with default settings to plot the genetic relationship matrix data.

## Additional information

**Accession codes:** The raw and analysed RNA-seq data have been deposited in the GEO database with accession code GSE59411.

**How to cite this article:** Bou Sleiman, M. S. *et al*. Genetic, molecular and physiological basis of variation in *Drosophila* gut immunocompetence. *Nat. Commun.* 6:7829 doi: 10.1038/ncomms8829 (2015).

## Supplementary Material

Supplementary Information Supplementary Figures 1-8, Supplementary Tables 1-6 and Supplementary References

Supplementary Data 1 Differential expression analysis between all challenged and all unchallenged samples.

Supplementary Data 2 Analysis of genes differentially expressed in resistant *versus* susceptible lines.

Supplementary Data 3 Modulated modularity clustering modules.
