# Supplementary material for: Genetic, molecular and physiological basis of variation in Drosophila gut immunocompetence
Source: Nat Commun. 2015 Jul 27;6:7829. doi: 10.1038/ncomms8829 (PMC4525169; doi:10.1038/ncomms8829)
Supplement: Supplementary Information — Supplementary Figures 1-8, Supplementary Tables 1-6 and Supplementary References [file ncomms8829-s1.pdf]

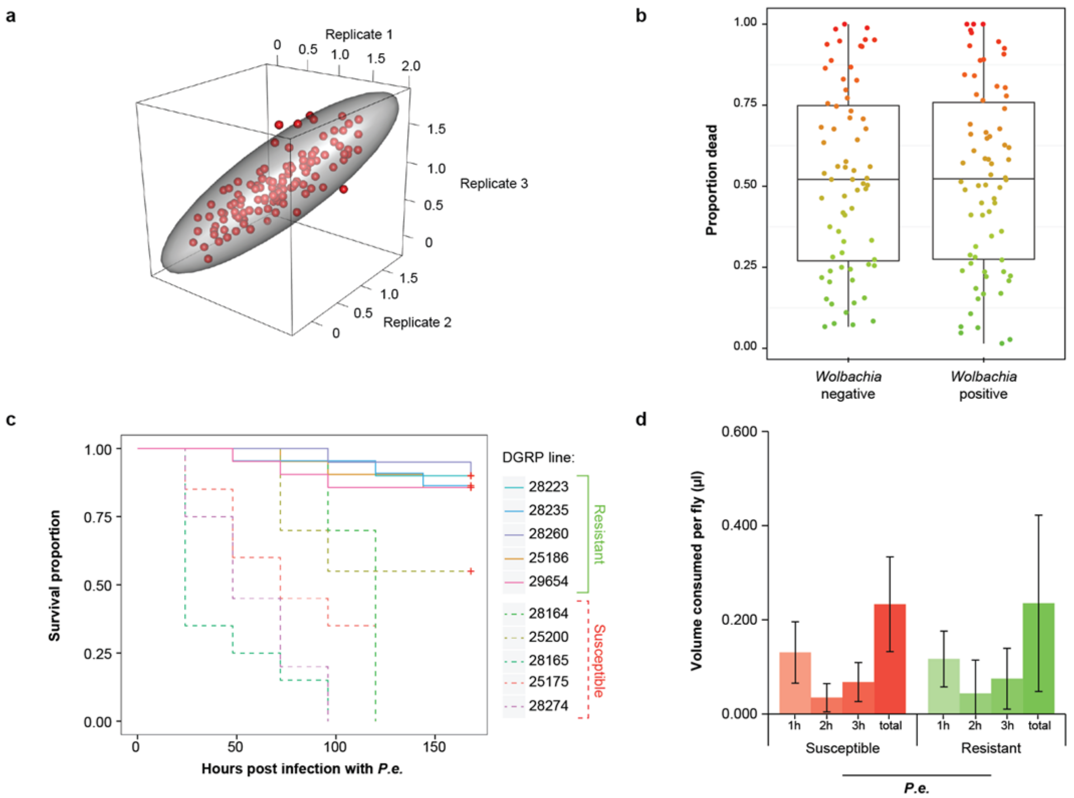

Supplementary Figure 1. Feeding behaviour, *Wolbachia*, and microbiota do not have a major influence on susceptibility to enteric infection. (a) The three survival experiment repeats represented in a three-dimensional scatter plot showing proportion deaths (after angular transformation) three days post infection. Each red point is a DGRP line and the confidence ellipsoid is in grey. (**b)** *Wolbachia* infection status does not correlate with susceptibility (Nested ANOVA p=0.51 for *Wolbachia* status effect on survival). 68 lines and 70 lines are *Wolbachia* negative and positive, respectively. (**c**) Flies that were either resistant or susceptible to enteric infection in non-axenic conditions were infected with *P.e.* under axenic conditions. Absence of the endogenous intestinal microbiota does not alter the relative susceptibility of the DGRP flies. (**d**) A Capillary Feeder (CAFE) assay shows that susceptible and resistant DGRP flies ingest a comparable volume of bacteria during the first three hours post *P.e.* infection.

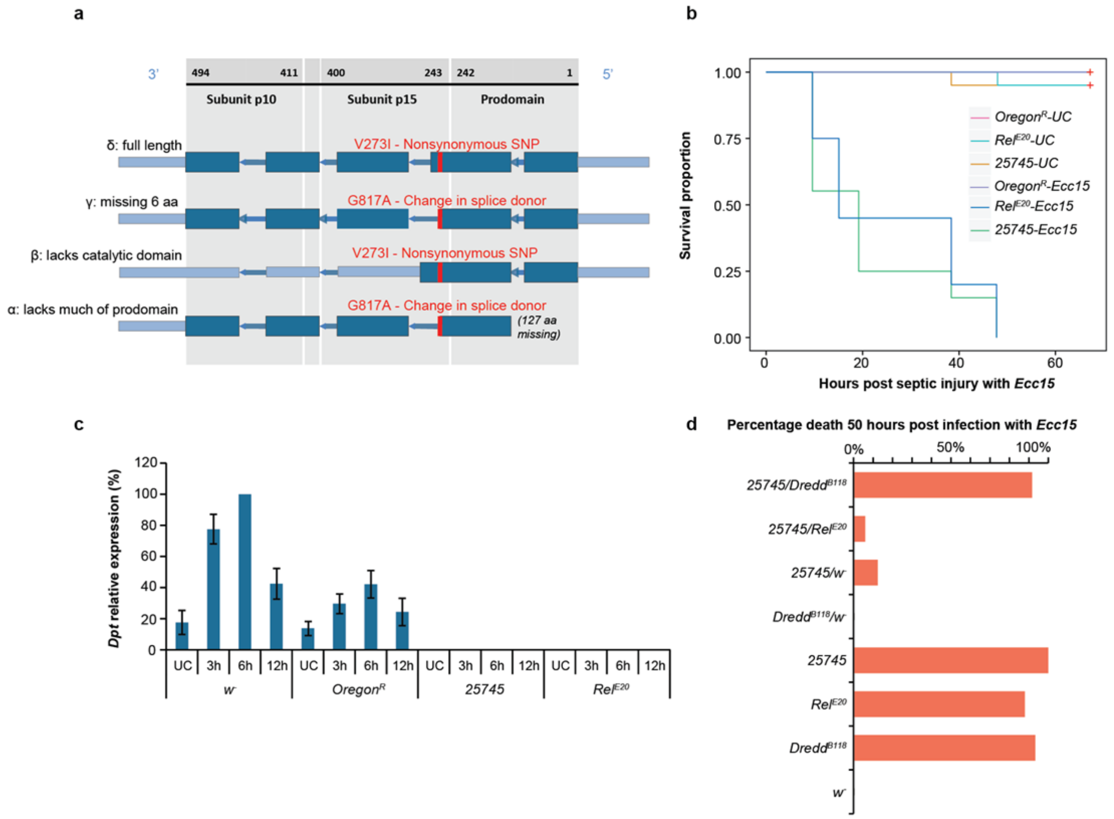

Supplementary Figure 2. Identification of a loss of function mutation in the *dredd* locus in one DGRP line. (a) Four isoforms related to the *dredd* gene have been previously described in [1]. γ and δ isoforms differ only by six amino acids. The α isoform lacks much of its prodomain and the β isoform lacks its catalytic domain. One SNP has been identified in the *dredd* locus of the DGRP line *#25745*, causing a change in the splicing donor site (G817A) in the α and γ mRNA, or an amino acid change (V273I) in δ and β isoforms. The light blue colours represent non-coding regions, the dark blue ones depict exons. **(b)** Survival analysis of females systemically infected with *Ecc15* shows a lower survival rate of the *#25745* line and *relish* mutant (Rel^{E20}) compared to controls (Log-Rank test p<0.05). **(c)** RT-qPCR experiments show that, similar to *relish* mutants, the *#25745* line systemically infected with *Ecc15* has no detectable *diptericin* (*Dpt*) expression as shown in w^- and $Oregon^R$ control flies. Data is normalized to 100% ± S.D. *w-* flies consistently had the highest level of *Dpt* induction (100%), hence the missing error bar. **(d)** Percentage of dead female flies 50 hours post *Ecc15* systemic infection is monitored. Only complementation of *#25745* line with a *dredd* mutant line fails to restore the wild-type survival, revealing that the identified SNP in the *dredd* gene is the causal locus of susceptibility to bacterial infection. Data presented in **b** and **c** are derived from three independent replicates.

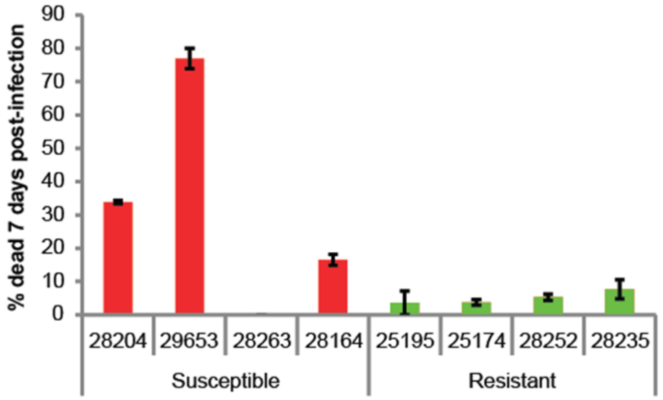

Supplementary Figure 3. Lines resistant to *P. entomophila* are also resistant to a clinical isolate of *Pseudomonas aeruginosa*. Bar chart showing the proportion of dead flies after 7 days post-infection (± s.d.; three biological replicates). The lines in the susceptible and resistant classes were identified based on their susceptibility to *P. entomophila* oral infection.

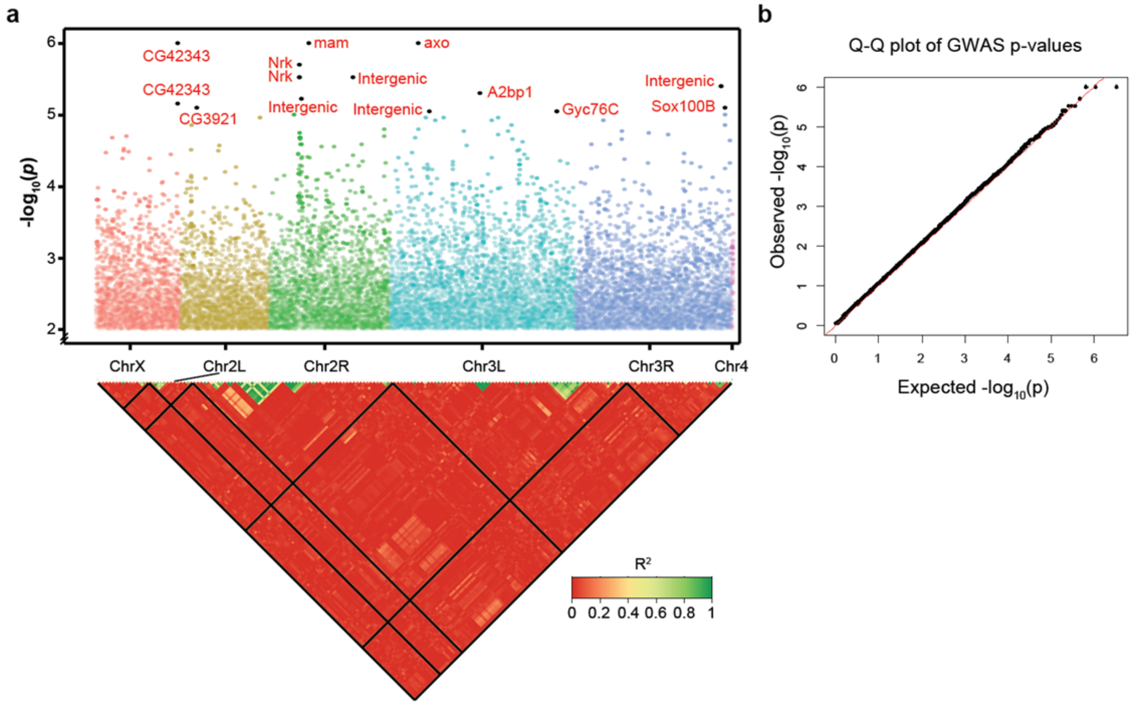

Supplementary Figure 4. Different statistical approaches yield highly similar GWAS top hits. (a) Above: Manhattan plot of the *p*-values (y-axis) for the association between genomic variants in DGRP lines and *P.e.* susceptibility. The x-axis represents the genomic location. A linear model was implemented in PLINK using angular-transformed proportion death at day 3 as phenotype. **Below:** heatmap of pairwise LD between all SNPs with a p-value < 10^{-4} (n=188). **(b)** Q-Q plot of the linear association.

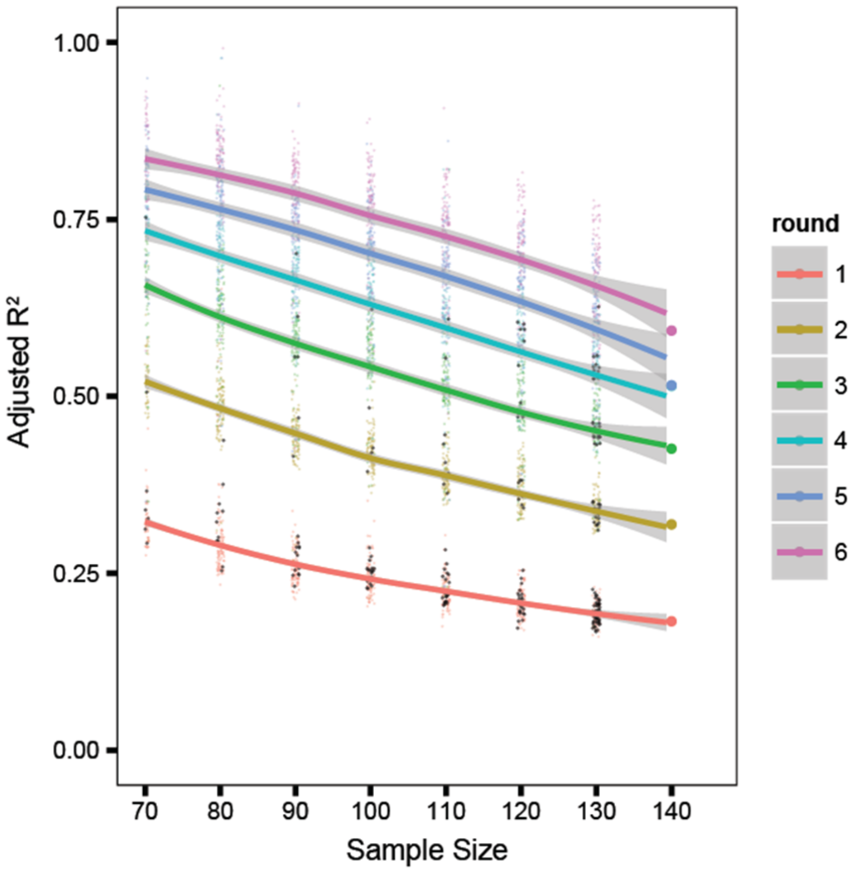

Supplementary Figure 5. Illustration of the Beavis effect. A plot of the adjusted R^2 values obtained through random sampling of lines with different sample sizes (100 random samples per size group) and multi-SNP association (six rounds of association). The curves are loess fits with 95% confidence interval, and black points correspond to SNPs that have been identified in the full population.

a

tub-GAL80ts;da-Gal4,Dpt-lacZ

UAS-RNAi	GWAS *p*-value	Dpt-LacZ induction
Nrk	3.60E-06	-
cv-c	7.28E-06	++
CG10147	7.32E-06	+
mam	9.10E-06	-
5-HT1A	1.85E-05	++
Gyc76C	1.86E-05	-
control	-	+

b

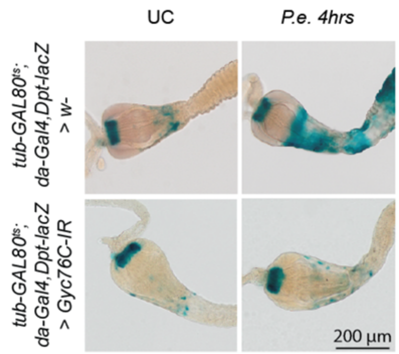

Supplementary Figure 6. Validation of candidate genes. (a) UAS-RNAi lines screened for an effect of *dipt-LacZ* reporter induction under a ubiquitous driver (*da-gal4*). "+" and "-" indicate higher and lower induction than control (w^{1118}), respectively, and the number of +'s scales with the extent of induction. (**b**) Knock-down of the top GWAS candidate gene, *Gyc76C*, using *da-gal4* highly reduces the induction of the immune activation reporter *Dpt-lacZ* in the gut as revealed with X-Gal staining.

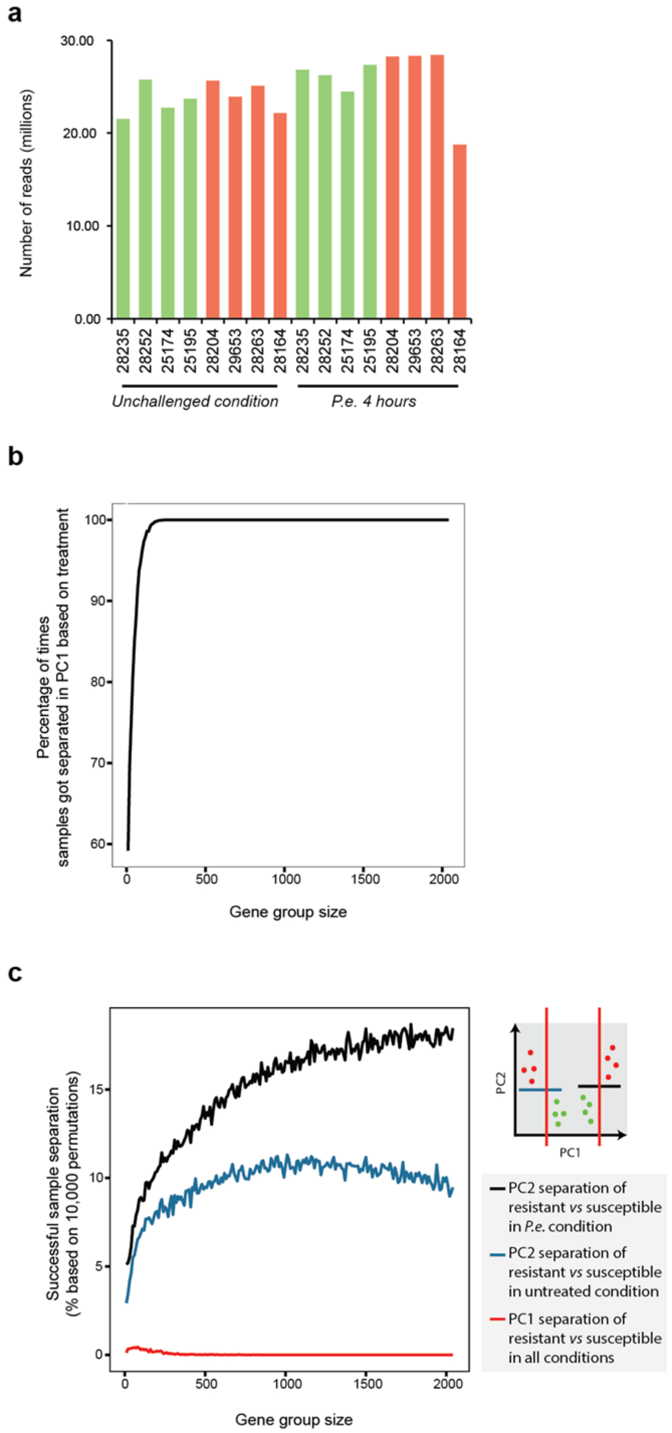

Supplementary Figure 7. Permutations of random sampling followed by PCA of the RNA-seq data. (a) RNA-seq library sizes of the 16 samples used in the study. **(b)** Random sampling of gene groups with sizes ranging from 10 to 2000 (10,000 permutations per group size), followed by PCA analysis on their gene expression levels revealed that treated and untreated samples are always separated by the first PC for groups greater than 250. **(c)** The same random sampling and PCA as in **b**, but with different separation criteria (see legend).

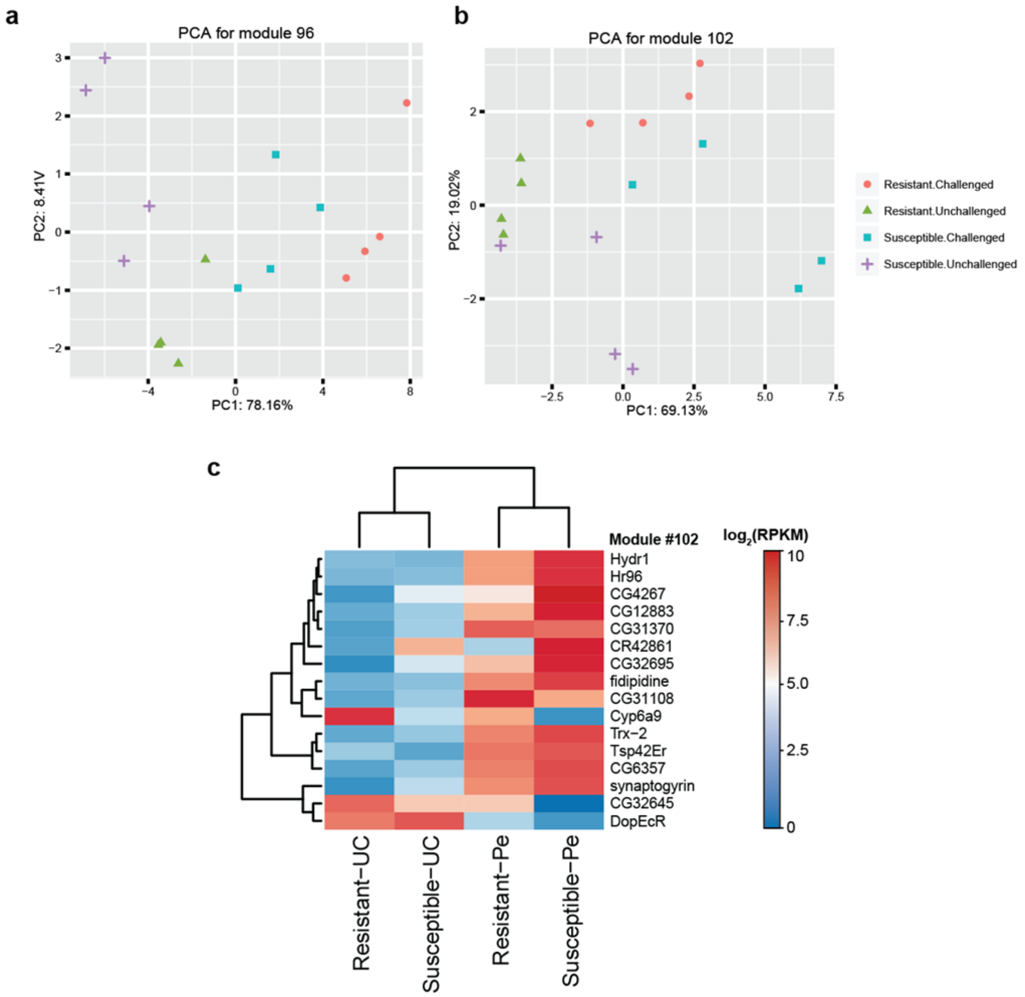

Supplementary Figure 8. Principal component analysis of modules (a):#96 and (b)#102. (c) Heatmap of average expression levels of genes in module #102 by susceptibility/treatment (unchallenged = UC or infected = *Pe*) class.

Supplementary Table 1. Percentage death of tested DGRP lines 3 days post-infection with *Pseudomonas entomophila*

DGRP#	Bloomington stock number	Percentage dead at day 3
DGRP-897	28260	0.50%
DGRP-802	28235	1.80%
DGRP-320	29654	4.30%
DGRP-738	28223	4.80%
DGRP-208	25174	7.60%
DGRP-857	28252	4.50%
DGRP-486	25195	10.00%
DGRP-129	28141	7.20%
DGRP-313	25180	7.20%
DGRP-360	25186	2.40%
DGRP-303	25176	10.50%
DGRP-142	28144	9.70%
DGRP-907	28262	15.00%
DGRP-217	28154	12.70%
DGRP-801	28234	16.30%
DGRP-379	25189	20.80%
DGRP-158	28147	24.40%
DGRP-237	28160	16.60%
DGRP-441	28198	16.90%
DGRP-440	28197	22.00%
DGRP-426	28196	11.70%
DGRP-399	25192	24.70%
DGRP-321	29655	23.10%
DGRP-894	28259	23.10%
DGRP-45	28128	20.30%
DGRP-335	25183	20.70%
DGRP-307	25179	25.20%
DGRP-837	28246	27.20%
DGRP-91	28136	21.50%
DGRP-161	28148	25.80%
DGRP-705	25744	35.50%
DGRP-377	28186	29.30%
DGRP-822	28244	22.00%
DGRP-804	28236	30.90%
DGRP-861	28253	21.80%
DGRP-799	25207	32.70%
DGRP-812	28240	37.40%
DGRP-356	28178	23.10%
DGRP-370	28182	27.90%
DGRP-373	28184	40.80%
DGRP-437	25194	33.50%
DGRP-195	28153	26.30%
DGRP-406	29657	24.10%
DGRP-318	28168	45.00%
DGRP-136	28142	34.30%
DGRP-41	28126	42.70%
DGRP-461	28200	27.70%
DGRP-805	28237	25.70%
DGRP-517	25197	46.70%
DGRP-563	28211	46.10%
DGRP-352	28177	53.60%
DGRP-75	28132	51.40%
DGRP-315	25181	50.40%
DGRP-642	28216	49.30%
DGRP-737	28222	50.10%
DGRP-371	28183	56.30%
DGRP-391	25191	48.80%
DGRP-859	25210	41.90%
DGRP-256	28162	44.90%
DGRP-42	28127	51.00%
DGRP-855	28251	48.90%
DGRP-362	25187	44.50%

DGRP-884	28256	52.00%
DGRP-350	28176	52.20%
DGRP-513	29659	41.00%
DGRP-808	28238	33.30%
DGRP-177	28150	52.70%
DGRP-786	25206	57.30%
DGRP-783	28230	49.70%
DGRP-375	25188	52.60%
DGRP-374	28185	63.00%
DGRP-381	28188	55.00%
DGRP-508	28205	54.10%
DGRP-820	25208	63.30%
DGRP-832	28245	52.60%
DGRP-57	29652	55.90%
DGRP-83	28134	68.30%
DGRP-492	28203	41.60%
DGRP-589	28213	40.90%
DGRP-239	28161	57.70%
DGRP-309	28166	68.20%
DGRP-796	28233	65.00%
DGRP-427	25193	71.10%
DGRP-304	25177	70.40%
DGRP-555	25198	72.10%
DGRP-26	28123	72.90%
DGRP-324	25182	57.30%
DGRP-491	28202	77.30%
DGRP-310	28276	50.40%
DGRP-712	25201	64.90%
DGRP-892	28258	57.40%
DGRP-380	25190	77.70%
DGRP-332	28171	15.20%
DGRP-409	28278	59.50%
DGRP-595	28215	82.80%
DGRP-776	28229	68.40%
DGRP-338	28173	59.20%
DGRP-392	28194	77.60%
DGRP-181	28151	58.60%
DGRP-509	28206	63.90%
DGRP-730	25202	80.20%
DGRP-732	25203	82.90%
DGRP-233	28159	77.70%
DGRP-109	28140	87.00%
DGRP-176	28149	85.10%
DGRP-911	28264	68.80%
DGRP-358	25185	88.10%
DGRP-365	25445	80.70%
DGRP-879	28254	79.20%
DGRP-28	28124	86.80%
DGRP-359	28179	95.30%
DGRP-531	28207	67.80%
DGRP-790	28232	77.90%
DGRP-502	28204	94.90%
DGRP-228	28157	88.80%
DGRP-405	29656	93.80%
DGRP-153	28146	84.40%
DGRP-639	25199	96.60%
DGRP-818	28241	93.00%
DGRP-882	28255	95.70%
DGRP-714	25745	98.30%
DGRP-535	28208	98.20%
DGRP-38	28125	89.40%
DGRP-386	28192	96.00%
DGRP-890	28257	93.40%
DGRP-761	28227	66.80%
DGRP-138	28143	80.50%
DGRP-721	28220	93.60%
DGRP-101	28138	96.80%
DGRP-40	29651	99.40%
DGRP-229	29653	99.50%
DGRP-908	28263	99.60%

DGRP-280	28164	100.00%
DGRP-287	28165	100.00%
DGRP-301	25175	100.00%
DGRP-85	28274	100.00%
DGRP-227	28156	65.80%
DGRP-707	25200	100.00%
DGRP-765	25204	64.40%
DGRP-774	25205	93.50%

Supplementary Table 2. Analyses of variance for diallel survival data (after angular transformation).

Effect	df	Mean Square	F	*P*
ANOVA on male/female effects due to resistant/susceptibility category				
Male resistance category	1	5.477	271.819	<0.001
Female resistance category	1	2.266	38.159	0.001
Male strain (nested within category)	6	.020	.275	0.946
Female strain (nested within category)	6	.060	.807	0.570
Male category x Female category	1	.814	11.025	0.002
Male strain x Female strain	46	.075	2.813	<0.001
Replication	63	1.715	64.183	<0.001
Diallel ANOVA testing for general and specific combining ability				
General combining ability	7	0.264	6.682	<0.001
Specific combining ability	28	0.215	5.444	<0.001
Reciprocal	28	0.166	4.184	<0.001
Maternal	7	0.13	0.735	0.64287
Maternal interaction	21	0.177	4.481	<0.001
Error	63	0.04		

The first ANOVA tests for effects due to male/female strain and susceptibility class (susceptible or resistant) and their interactions on survival. Strain was nested within the resistant or susceptible categories and treated as a random variable. The second ANOVA represents the diallel analysis according to Griffing (1956)[2] testing for general combining ability (additive effects and their interactions) and specific combining ability (dominance effects and their interactions) as well as effects due to reciprocal differences in the crosses, maternal contributions, and their interactions.

Model for ANOVA: $Y_{ijklm} = \mu + m_i + f_j + s_{k(i)} + t_{l(j)} + m_i f_j + s_{k(i)} t_{l(j)} + e_{ijklm}$ where μ is the population mean, m_i is the *i*th male category, f_j is the *j*th female category, $s_{k(i)}$ is the *k*th male strain within the male category, $t_{l(j)}$ is the *l*th female strain within the female category and e_{ijklm} is the residual. Strain within categories are random, other terms apart from replication are fixed.

Model for diallel analysis: $Y_{ijklm} = \mu + g_i(g_j) + s_{ij} + r_{ij} + m_i + n_{ij} + e_{ijk}$ where μ is the population mean, $g_i(g_j)$ is the general combining ability for the *i*th (*j*th) parents, *sij* is the special combining ability for the cross between the *i*th and *j*th parents, r_{ij} is the reciprocal effect, m_i is the maternal effect, n_{ij} is the interaction of the *i*th maternal effect with the *j*th parent, and e_{ijk} is the error term. The analysis follows Method 1 (parents and reciprocal F1s measured) under Model 1 of Griffing (1956)[2] with maternal terms added [3,4].

Supplementary Table 3. Summary of top QTLs obtained in common between parametric and non-parametric association studies.

Genomic location	Variant annotation	Kruskal-Wallis p value[a]	PLINK empirical p-value[b]	Number of permutations[c]	R^{2} [d]
Chr2R:9048826	Nrk (intron)	3.60E-06	3.00E-06	1000000	0.14
Chr2R:9048897	Nrk (exon V306G)	3.60E-06	3.00E-06	1000000	0.14
Chr2R:9048840	Nrk (intron)	4.40E-06	2.00E-06	1000000	0.14
Chr3R:26527712	Intergenic - Pka-C2(dist=4852),CG31010(dist=2770)	4.93E-06	4.00E-06	1000000	0.15
Chr3R:26527703	Intergenic - Pka-C2(dist=4843),CG31010(dist=2779)	4.93E-06	4.00E-06	1000000	0.15
Chr2L:3172873	Intergenic - CG34406(dist=123);CG31698(dist=411)	6.83E-06	3.10E-05	1000000	0.12
Chr3R:10229978	cv-c (intron)	7.28E-06	1.70E-05	1000000	0.13
Chr3L:6480167	CG10147 (exon, synonymous)	7.32E-06	1.20E-05	1000000	0.13
Chr2R:9892328	mam (intron), CG30482 (exon)	9.10E-06	1.00E-06	1000000	0.16
Chr3L:6076155	Intergenic - CG6619(dist=1520),CG13293(dist=4214)	1.35E-05	5.85E-05	752247	0.11
Chr3R:10227723	cv-c (intron)	1.36E-05	2.20E-05	1000000	0.14
ChrX:21324090	CG42343 (intron)	1.41E-05	1.00E-06	1000000	0.19
Chr3L:9361423	CG4452 (intron)	1.45E-05	1.70E-05	1000000	0.12
Chr3L:10570926	A2bp1 (intron)	1.55E-05	5.00E-06	1000000	0.17
Chr2R:19991068	enok (exon, synonymous)	1.57E-05	1.60E-05	1000000	0.10
Chr2R:14967476	5-HT1A (intron)	1.85E-05	5.11E-05	861138	0.10
Chr3L:19769316	CG42637,Gyc76C (intron)	1.86E-05	9.00E-06	1000000	0.15
ChrX:4208879	mei-9 (3' UTR)	1.89E-05	3.40E-05	1000000	0.10
Chr2L:3794426	CG3921 (exon, synonymous)	1.90E-05	8.00E-06	1000000	0.15
Chr2R:10603181	Intergenic - mspo(dist=2055),CG12865(dist=23043)	1.94E-05	8.09E-05	544000	0.11
Chr2R:8613576	CG42663 (intron)	2.76E-05	1.00E-05	1000000	0.16
Chr2R:8613586	CG42663 (intron)	4.34E-05	1.00E-05	1000000	0.16
Chr2R:16288827	Intergenic - CG11192(dist=46270),CG12484(dist=23014)	5.18E-05	3.00E-06	1000000	0.15
Chr3R:5045687	pum (intron)	7.31E-05	5.67E-05	776000	0.11
Chr2R:12715416	CG34459(dist=1264), CG34460(dist=1013)	8.12E-05	2.80E-05	1000000	0.08
ChrX:12947763	CG12715 (exon, synonymous)	9.30E-05	1.48E-04	298402	0.10
Chr2L:8635001	Sema-1a (intron)	2.25E-04	2.70E-05	1000000	0.11

[a] Non-parametric association p-value

[b] Empirical p-value after adaptive permutation as implemented in PLINK[5]

[c] Number of permutations performed for each SNP

[d] Linear model R^2 for single SNPs

Supplementary Table 4. Additive multiple-SNP model results

GWAS Round	Top SNP	Coefficient	p-value	Adjusted R^2
1	Chr3L:4668479	-0.3251	1.46E-07	0.18
2	Chr2R:9892328	-0.2683	5.64E-07	0.32
3	Chr2L:3355610	0.4201	1.54E-06	0.43
4	Chr3L:13828661	-0.3401	1.90E-06	0.51
5	Chr2L:3355661	-0.4183	7.30E-07	0.52
6	Chr2L:2836880	0.3875	1.84E-06	0.59
7	Chr2L:2836903	-0.3888	2.00E-06	0.58
8	Chr3L:15759197	-0.1970	5.79E-06	0.64
9	Chr3R:15278253	0.1810	5.08E-06	0.69
10	Chr3R:15278255	-0.1810	5.08E-06	0.69
11	Chr3L:9600645	0.1600	5.35E-06	0.74
12	Chr2L:12809795	0.1499	1.25E-05	0.78
13	Chr3L:9680631	-0.1815	3.66E-06	0.83
14	Chr3R:9554355	-0.1739	2.10E-06	0.87
15	Chr3R:9554381	-0.1739	2.10E-06	0.87
16	Chr2L:18589931	0.1971	3.59E-05	0.90
17	Chr2R:10000342	0.1574	0.000117	0.91
18	Chr3L:3312435	-0.1575	0.000171	0.93
19	Chr2R:16922817	-0.1419	5.25E-05	0.94
20	ChrX:20010029	-0.1835	4.19E-05	0.95

Successive iterations of the GWAS were performed using a linear model of the form $Y = \mu + SNP_1 + SNP_2 + SNP_3 + \ldots\ SNP_N + \epsilon$, where $SNP_1, SNP_2, SNP_3, \ldots,\ SNP_N$, are the most significant SNPs fitted in succession as in Harbison et al., 2013[6]. In short, for each round a GWAS is performed and the SNP with the most significant QTL is recorded, which is then incorporated in the linear model of the next round.

Supplementary Table 5. Multiple-SNP regression for SNPs in module #96

geneID	GWAS p-value	snpID	Estimate	Std. Error	t	value
-	-	(Intercept)	0.33911	0.23743	1.428	0.15611
eas	1.40E-03	ChrX:16175381	0.13048	0.05661	2.305	0.02309
rev7	6.54E-01	Chr3R:1414703	0.13655	0.11685	1.169	0.24513
CG33158	5.54E-05	Chr3L:16415271	-0.22319	0.06629	-3.367	0.00105
Cyp6d2	1.11E-02	Chr2R:18540150	-0.10239	0.08927	-1.147	0.25393
CG10827	5.38E-03	Chr3R:16832600	-0.10546	0.07278	-1.449	0.15022
CG32669	7.07E-02	ChrX:10737211	0.06322	0.05821	1.086	0.27986
Gs2	4.12E-02	ChrX:11322919	-0.0274	0.06418	-0.427	0.67023
CG3625	8.59E-03	Chr2L:284365	-0.23906	0.0887	-2.695	0.00816
GstD10	8.17E-02	Chr3R:8191081	-0.02762	0.06186	-0.446	0.65618
yip2	4.24E-02	Chr2L:9915438	0.16849	0.12498	1.348	0.18044
SMC2	9.89E-02	Chr2R:10736815	-0.13882	0.10309	-1.347	0.18095
lectin-37Da	1.71E-02	Chr2L:19418365	-0.14842	0.1091	-1.36	0.17654
Dgp-1	5.59E-02	Chr2R:14057889	0.02383	0.10814	0.22	0.82603
GstD9	1.25E-01	Chr3R:8192383	0.20098	0.10987	1.829	0.07014
Ugt36Ba	1.01E-01	Chr2L:16794249	0.05927	0.06907	0.858	0.39268
CG11309	4.37E-02	Chr3L:21297350	0.08747	0.08353	1.047	0.29735
GstD1	1.42E-01	Chr3R:8194750	0.01066	0.1343	0.079	0.93691
gukh	3.36E-03	Chr3R:14827525	0.19141	0.09755	1.962	0.05233
Sodh-2	3.97E-02	Chr3R:6702928	0.06843	0.11717	0.584	0.56044
RPA3	1.28E-01	ChrX:11615178	0.06898	0.06521	1.058	0.29256

Residual standard error: 0.3029 on 108 degrees of freedom
(11 observations deleted due to missingness)

Adjusted R-squared: 0.2961
F-statistic: 3.693 on 20 and 108 DF, p-value: 5.569e-06

One SNP with the lowest GWAS p-value in the GWAS was chosen for each of the 20 genes in the module. The 20 SNPs were fitted simultaneously in a linear model of the form
$Y = \mu + SNP_1 + SNP_2 + SNP_3 + \ldots SNP_{20} + \epsilon$.

Supplementary Table 6. List of primer sequences used in the study

Target	Forward primer	Reverse primer
diptericin	ACCGCAGTACCCACTCAATC	CACACCTTCTGGTGACCCTG
RpL32	GACGCTTCAAGGGACAGTATCTG	AAACGCGGTTCTGCATGAG
Gyc76C	AAACATCGGATGAGCAGGCA	GTGTAGTCGCAGCCACAGAT
monalysin	CTGGGTAATGGCCGACAAGT	ACAGAATGTGACGACCACCC

Supplementary References

1. Di Fruscio M, Styhler S, Wikholm E, Boulanger M-C, Lasko P, Richard S. kep1 interacts genetically with dredd/Caspase-8, and kep1 mutants alter the balance of dredd isoforms. *Proceedings of the National Academy of Sciences* **100**, 1814-1819 (2003).

2. Griffing B. Concept of General and Specific Combining Ability in Relation to Diallel Crossing Systems. *Australian Journal of Biological Sciences* **9**, 463-493 (1956).

3. Cockerham CC, Weir BS. Quadratic Analyses of Reciprocal Crosses. *Biometrics* **33**, 187-203 (1977).

4. Kaushik LS, Puri PD. A Study of Maternal and Maternal Interaction Effects in Diallel Crosses. *Biometrical Journal* **26**, 771-777 (1984).

5. Purcell S*, et al.* PLINK: A Tool Set for Whole-Genome Association and Population-Based Linkage Analyses. *American Journal of Human Genetics* **81**, 559-575 (2007).

6. Harbison S, McCoy L, Mackay T. Genome-wide association study of sleep in Drosophila melanogaster. *BMC Genomics* **14**, 281 (2013).
